# Skin Diseases and Syphilis

**Published:** 1898-05

**Authors:** 


### *SELECTIONS AND ABSTRACTS.*

### SKIN DISEASES AND SYPHILIS.

EDITED BY DR. M. B. HUTCHINS.

### DIFFUSE SCLERODERMA.

Dr. Wm. Osler, of Baltimore, has an article upon this "hardening, stiffening, contracting" disease of the skin, in the February and March, 1898, *Journal of Cutaneous and Genito-Urinary Diseases.* He dwells upon the diagnosis, gives some case histories, and discusses the treatment of the disease with thyroid gland extract. Thyroid extract first came into use in the treatment of myxedema—in the absence of normality in the thyroid gland of the patient. The substance was useful in that condition. Dr. Osler's experience with the drug was that which was to have been expected—the treatment was of no benefit. The extract has been used in various conditions, empirically, and, for the most part, unsuccessfully.

Cases of Addison's disease—a bronzing of the skin, supposedly due, in the majority of cases, to disease of the supra-venal capsules—have been treated with extract of the supra-venals of animals, and probably were benefited.

In cases due to deficiency of certain glands in the body, it is reasonable to suppose that extracts from the same glands in the lower animals would be of benefit; but it does not follow that the purely empirical use of these extracts in different conditions would do good.

### MERCURIAL ERUPTIONS AND ERUPTIONS FROM MERCURIAL INUNCTION.

*The Journal of Cutaneous and Genito-Urinary Diseases,* February, 1898, quotes Rosenthal (*Wien Med. Wochenschr.,* 1897) and Stopford Taylor (*Brit. Journ. Derm.,* 1897, p. 346). Symptoms vary from a never suppurating folliculitis to various urticarial, erythematous and even hemorrhagic eruptions. Eczema, purpura and other skin symptoms may occur. Severe general symptoms and death may follow. Eruption may spread from the point of application of the mercury or it may result, in various places, from the internal use of the drug in any form. Even inhalation may be followed by an eruption, with great scaling. Idiosyncrasy does not play so important a part as does the kind of drug, the condition of the kidneys, mental disorders and change to a tropical climate.

The list of drugs capable of producing skin eruptions would almost index the pharmacopœia. Those which produce a characteristic eruption are few. It is a toxic action referred to the skin, and is to be compared to that of various other substances which produce some form of skin eruption. The internal use of mercury is less likely to produce skin manifestations than the external, owing to its elimination through the intestines.

---

### A CASE OF DISSEMINATED CARCINOMA OF THE TRUNK.

Dr. Chas. W. Allen presented this case before the New York Dermatological Society, Nov., 1897 (*Journ. Cut. and G. U. Dis.*, March, 1898): In 1891 Dr. Allen amputated the right breast of the patient, an old lady, including the axillary tissues and parts of the pectoral muscles. The patient remained well for about four years. Then a nodule appeared in the line of incision. When shown, the anterior, lateral and posterior aspects of the right chest wall and the left breast were involved. Nodules had also developed over the lumbar region.

This case demonstrates the uncertainty as to prognosis in cancer of the breast. This patient either had the carcinomatous tendency, the disease redeveloping *de novo* in the scar, or some cells were left in the operation, or implanted from elsewhere, and then remained quiescent for some years. It is questionable if the first of these suppositions is not the more nearly correct.

---

### ARSENICAL PIGMENTATION.

Dr. W. A. Hardaway, of St. Louis, has a report of two cases, and some remarks upon this condition, in the April, 1898, *Journ. of*

*Cutan. and G. U. Diseases.* Pigmentation of the skin after prolonged use—internally—of arsenic is well known. Congestive or inflammatory conditions of the skin followed by the pigmentation, due to arsenic, is not so commonly recognized. The doctor has some doubt whether this pigmentation is ever the first symptom. The discoloration is not comparable to argysia, which is due to the silver deposit in the skin. His cases showed the erythematous symptoms first, and the pigmentation which followed was limited to the previously affected spots.

Even temporary congestion or inflammation of the skin is often followed by an increased deposition of pigment. The site of the local use of a sinapism often remains clearly defined, through pigmentation, for a long time, and erythema or inflammation from an internal cause can act in the same way.

---

### CANCER OF THE URETHRA.

The above Journal also quotes cases of Drs. Binaud and Chavannaz, reported to the French Association of Genito-Urinary Surgeons in October. It was a "curious form." Patient was a man of forty-four. The penis was deformed. The posterior was indurated and appeared as if in erection ; the anterior portion, with the glans, was normal. The patient died, and autopsy disclosed encephaloid cancer of the back part of the anterior urethra, a part of one corpus cavernosum, prostate and deep urethra.

